# Targeting Oxidative Stress in Insulin-Resistant Postmenopausal Women: Effects of Resveratrol and Vitamin C on Oxidative Damage and Antioxidant Defenses in a Randomized Clinical Trial

**DOI:** 10.3390/antiox15081028

**Published:** 2026-08-18

**Authors:** Enrique Reyes-Muñoz, Arturo Arellano-Eguiluz, Guillermo F. Ortiz-Luna, Alberto M. Guzmán-Grenfell, Arturo Flores-Pliego, Aurora Espejel-Nuñez, Sandra B. Parra-Hernández, Elizabeth García-Gómez, José Romo-Yañez, Araceli Montoya-Estrada

**Affiliations:** 1 Coordination of Gynecological and Perinatal Endocrinology, National Institute of Perinatology, Ministry of Health, Mexico City 11000, Mexico; enrique.reyes@inper.gob.mx; 2 Coordination of Peri and Postmenopause, National Institute of Perinatology, Ministry of Health, Mexico City 11000, Mexico; arturo.arellano@inper.edu.mx (A.A.-E.); guillermo.ortiz@inper.gob.mx (G.F.O.-L.); 3 Department of Immunobiochemistry, National Institute of Perinatology, Ministry of Health, Mexico City 11000, Mexico; aguzmangrenfell@yahoo.com.mx (A.M.G.-G.); arturo.flores@inper.gob.mx (A.F.-P.); aurora.espejel@inper.gob.mx (A.E.-N.); sandrabpahdz@gmail.com (S.B.P.-H.); 4 Council of Science, Humanities, Technologies and Innovation (SECIHTI)-Human Reproduction Research Unit, National Institute of Perinatology—Faculty of Chemistry, National Autonomous University of Mexico, Mexico City 11000, Mexico; egarciag@secihti.mx

**Keywords:** postmenopause, antioxidants, polyphenol, catalase, glutathione reductase, 8-isoprostane, insulin resistance

## Abstract

Women experience significant changes throughout their lives, particularly during the transition from reproductive age to postmenopause, marked by hormonal imbalance and reduced estrogen production. This stage is associated with increased oxidative stress, which can contribute to cardiovascular issues and insulin resistance. This study aimed to assess the effects of resveratrol and vitamin C supplementation on 8-Isoprostane, a marker of oxidative stress, and on DNA damage, as indicated by 8-hidroxi-2′-desoxiguanosina (8-Oxo-dG), in postmenopausal women with insulin resistance, and to evaluate how these supplements modified antioxidant defense. A randomized, double-blind clinical trial was conducted. Plasma samples were analyzed to measure antioxidant enzyme activity, including superoxide dismutase (SOD), catalase (CAT), glutathione reductase (GR), and glutathione peroxidase (GPx), and to assess oxidative damage to lipids and DNA. Forty-two patients were recruited and randomly assigned to three treatment groups. Within-group analyses showed significant increases in CAT and GR activities and a reduction in plasma 8-isoprostane concentrations in the group receiving combined resveratrol and vitamin C supplementation, with increases of 12% in CAT activity (*p* = 0.008) and 84% in GR activity (*p* = 0.007), and a 34% reduction in 8-isoprostane concentrations (*p* = 0.01). However, no significant between-group differences or treatment × time interactions were observed. No significant changes were detected in 8-Oxo-dG concentrations. These preliminary findings suggest that antioxidant supplementation may influence selected markers of antioxidant defense and lipid oxidative damage in postmenopausal women with insulin resistance; however, the present study does not demonstrate the superiority of one supplementation regimen over the others.

## 1. Introduction

During postmenopause, there is a decrease in ovarian estrogen production, leading to reduced antioxidant defenses and, in turn, increased pro-oxidant generation, which affects women’s quality of life [1]. It has been reported that the increase in free radicals exceeds the body’s antioxidant defense mechanisms. Therefore, it would be important to compensate for the loss of estrogen with natural supplements that can provide electron-donor molecules to reduce free radicals [2].

The production of reactive oxygen species (ROS) is a natural and continuous process, and they participate in physiological functions within the body. The damage these compounds can cause depends on a delicate balance with the antioxidant systems that protect the cells [3]. Enzymatic antioxidants, such as superoxide dismutase (SOD), catalase (CAT), glutathione reductase (GR), glutathione peroxidase (GPx), and glutathione transferase; and non-enzymatic antioxidants, including ascorbic acid, glutathione, tocopherols, coenzyme Q, flavonoids (Resveratrol, quercetin), and carotenoids (lutein, lycopene). These antioxidant systems act coordinately to preserve cellular redox homeostasis and limit oxidative damage [4,5,6].

Because antioxidant enzymes constitute the first line of defense against species ROS, changes in their activity reflect the capacity of the endogenous antioxidant system to maintain redox homeostasis. Complementary assessment of 8-isoprostanes, one of the most reliable biomarkers of lipid peroxidation generated through the non-enzymatic oxidation of arachidonic acid, and 8-hydroxy-2′-deoxyguanosine (8-Oxo-dG), a widely accepted biomarker of oxidative DNA damage that reflects the balance between DNA oxidation and DNA repair, provides an integrated evaluation of oxidative stress at multiple biological targets [1,7,8].

During postmenopause, women experience a loss of estrogen and a decline in ovarian endocrine function, which affects antioxidant capacity [9]. This decline also increases components of metabolic syndrome, such as higher abdominal circumference, increased blood pressure, elevated serum cholesterol, low-density lipoprotein (LDL), and glucose levels. These changes are often associated with higher risks of various diseases, including cardiovascular disease, insulin resistance, diabetes, and osteoporosis. Lastly, this period can be physically and emotionally challenging for women [10]. According to Mexican data, life expectancy at birth is 79 years for women and 72.6 years for men. It is estimated that women spend nearly a third of their lives in a postmenopausal state. Based on the National Survey on Health and Aging in Mexico, 54.4% of the 25.9 million people aged 53 and over are postmenopausal women [10].

Therefore, we are interested in evaluating the effects of phytoestrogens such as resveratrol and antioxidants such as vitamin C during postmenopause. Resveratrol (3,5,4-trihydroxystilbene) is an antioxidant in the stilbene family. It is a natural phytoestrogen and a flavonoid [11,12]. Its structure consists of two phenolic rings connected by a double bond, which gives rise to cis and trans isomers. This chemical structure resembles synthetic estrogens such as diethylstilbestrol and 17-β-estradiol. Consequently, some studies have tested its ability to act as a phytoestrogen, as there is no evidence of toxicity in humans at doses up to 2 g per day [13]. It is also a natural polyphenol found in many plants and fruits, including peanuts, blackberries, blueberries, and especially grapes and red wine [14].

In postmenopause, decreased antioxidant defense activity contributes to the development of chronic degenerative diseases [15,16]. Resveratrol has been described as having several therapeutic benefits, including cardioprotective effects, lowering LDL cholesterol, increasing HDL levels, and improving blood pressure [17]. Evidence also indicates effects on the endocrine, circulatory, skeletal, and nervous systems, as well as prior effects on reproductive health or pregnancy. Additionally, resveratrol not only neutralizes free radicals and fights inflammation, but also inhibits fat accumulation in the liver, improves endothelial function, and reduces insulin resistance. Among other antioxidants, vitamin C (ascorbic acid) protects cells against free radical damage [18]. It benefits women during reproductive stages like peri- and postmenopause by preventing the estrogen decline associated with aging. It also has antioxidant properties, neutralizing free radicals and balancing oxidative stress. Similarly, it alleviates the severity of hot flashes by enhancing kidney function and increasing estrogen production. This, in turn, strengthens the antioxidant defense system [19]. To improve antioxidant capacity, several studies have been conducted in adult women with different antioxidant treatments, including α-tocopherol and retinol [19], which demonstrated high levels—markers of oxidative stress—and low levels of the antioxidant enzymes CAT and superoxide dismutase in women who did not incorporate these vitamins into their diet. The use of cruciferous vegetable extracts (broccoli and flaxseed) has also been tested, which reduces the risk of breast cancer [20].

Given the limited number of studies demonstrating the effectiveness of antioxidant supplementation in increasing total antioxidant capacity, reducing oxidative damage, and improving insulin resistance in postmenopausal women, we propose an antioxidant therapy combining resveratrol and vitamin C to achieve synergistic effects. Currently, there is no comprehensive evaluation panel for assessing antioxidant defense in postmenopausal Mexican women with insulin resistance; therefore, our goal is to investigate the impact of combined antioxidant therapy as a potential strategy to enhance antioxidant defense and reduce oxidative damage.

## 2. Materials and Methods

### 2.1. Study Design and Participants

The design and ethical approval of this randomized, double-blind clinical trial have been described in detail previously [19]. Briefly, the study was conducted at the Peri and Postmenopause Clinic of the Instituto Nacional de Perinatología (INPer) in Mexico City from January 2019 to March 2020 (ClinicalTrials.gov: NCT03090997). All participants signed written informed consent.

Women aged 50 to 60 years who were in early postmenopause, as defined by the STRAW criteria stages +1A, +1B, and +1C, and had insulin resistance (HOMA ≥ 2.5) were included, provided they had no use of metformin, bezafibrate, or statins within three months before enrollment and no current indication for hormone replacement therapy.

Exclusion criteria included women who were on anticoagulant therapy or taking any antioxidant supplements before study initiation. Also excluded were participants with diagnoses of diabetes mellitus, rheumatoid arthritis, systemic lupus erythematosus, any type of cancer, HIV infection, or kidney disease. Active smokers and individuals unwilling to provide informed consent were also excluded. Additionally, participants who did not achieve at least 80% adherence to the prescribed treatment were withdrawn from the study.

### 2.2. Randomization and Allocation Concealment

Details on the randomization, blinding procedures, and the composition of the antioxidant and placebo capsules used in this trial have been reported previously [19]. In brief, participants were assigned to one of three groups: (A) Resveratrol and placebo, (B) Resveratrol and vitamin C, or (C) Vitamin C and placebo. Treatments and placebos were identical, and allocation concealment was maintained using opaque envelopes. Both participants and researchers remained blinded until the study concluded (ClinicalTrials.gov: NCT03090997). Treatment allocation was concealed using sequentially numbered, opaque, sealed envelopes prepared before participant enrollment. After enrollment, each participant received the corresponding intervention packaged in identical opaque bottles. Both participants and investigators remained blinded throughout the study. The treatment codes were maintained exclusively by the institutional pharmacologist and were disclosed only after completion of data collection and statistical analyses. Participants were instructed not to consume any antioxidant supplements during the study and to continue their habitual diet.

### 2.3. Primary Endpoint

The primary goal was to assess 8-Isoprostane as a marker of oxidative stress, specifically produced by lipid peroxidation of arachidonic acid, and to assess DNA damage, as indicated by 8-Oxo-dG.

Blood samples were obtained by antecubital venipuncture into tubes containing 2 mM ethylenediaminetetraacetic acid (EDTA) (BD Vacutainer, Franklin Lakes, NJ, USA), in the morning after an overnight fast of 8–10 h at two time points: at baseline, before the initiation of supplementation, and after three months of intervention with resveratrol and placebo, resveratrol and vitamin C, or vitamin C and placebo. Plasma was separated by centrifugation at 3000 rpm for 15 min, aliquoted, and stored at −70 °C until biochemical analyses. At the end of the study, participants continued with their routine climacteric medical follow-up.

Clinical status was assessed, and measurements such as waist circumference and blood pressure, along with lab tests—including glucose, triglycerides, total cholesterol, HDL-C, and LDL-C—were recorded.

### 2.4. Secondary Outcomes

The secondary outcomes involved assessing how resveratrol and vitamin C supplements modified antioxidant defense in postmenopausal women.

### 2.5. Biochemical Analysis: Quantification of Oxidative Damage

#### 2.5.1. 8-Isoprostane ELISA

Produced by the non-enzymatic peroxidation of arachidonic acid in membrane phospholipids, accompanied by a decrease in absorbance at 412 nm, it is proportional to the amount of 8-isoprostane-AChe tracer and inversely proportional to the amount of free 8-isoprostane. Assays showed intra-assay coefficients of variation ranged from 6.4% to 20.0%, whereas the inter-assay coefficient of variation ranged from 9.6% to 24.3%. The assay has a sensitivity of approximately 2.7 pg/mL and a working range of 0.8–500 pg/mL. To evaluate 8-isoprostane in plasma, the guidelines of the Cayman Chemical 8-Isoprostane ELISA Kit (Ann Arbor, MI, USA) were followed.

#### 2.5.2. 8-Hydroxy-2′-Deoxyguanosine

8-Oxo-dG is produced almost exclusively by oxidative stress, with the primary attack occurring at the N7-C8 bond by oxidative radicals. It is a biomarker of oxidative DNA damage and oxidative stress and is measured by absorbance at 450 nm. Assays showed intra- and inter-assay coefficients of variation of <10%/<15%. The assay has an analytical sensitivity of approximately 0.125 ng/mL and a measurement range of 0.125–10 ng/mL. For 8-Oxo-dG, the procedure followed the R&D Systems, Inc. HT 8-Oxo-dG ELISA Kit II (Minneapolis, MN, USA).

### 2.6. Biochemical Analysis: Quantification of Antioxidant Defense

#### 2.6.1. Superoxide Dismutase (SOD)

SOD uses a tetrazolium salt to detect superoxide radicals (O_2_^–^) produced by xanthine oxidase and hypoxanthine. The decrease in absorbance at 450 nm is directly proportional to SOD activity in the sample. Assays showed intra- and inter-assay coefficients of variation of 3.2%/3.7%. For this method, the instructions for the Cayman Chemical Superoxide Dismutase Assay Kit (Ann Arbor, MI, USA) were followed.

#### 2.6.2. Catalase (CAT)

CAT activity is based on the reaction of the enzyme with methanol in the presence of an optimal concentration of H_2_O_2_. The decrease in absorbance at 540 nm is directly proportional to CAT activity in the sample. Assays showed intra- and inter-assay coefficients of variation of 3.8%/9.9%. For quantification, the procedure followed the Cayman Chemical Catalase Assay Kit (Ann Arbor, MI, USA).

#### 2.6.3. Glutathione Reductase (GR)

GR activity is measured by the oxidation of NADPH to NADP+, which decreases absorbance at 340 nm. This decrease is directly proportional to GR activity in the sample. Assays showed intra- and inter-assay coefficients of variation of 3.7%/9.3% respectively. To obtain GR results, we used the Cayman Chemical Glutathione Reductase Assay Kit (Ann Arbor, MI, USA).

#### 2.6.4. Glutathione Peroxidase (GPx)

The rate of this decrease is directly proportional to GPx activity in the sample at 340 nm. Assays showed intra- and inter-assay coefficients of variation of 5.7% and 7.2%, respectively. For GPx determination, the Cayman Chemical Glutathione Peroxidase Assay Kit (Ann Arbor, MI, USA) was used according to the manufacturer’s recommendations.

All experiments were performed in duplicate. Sample readings were obtained using a microplate reader (Synergy HT, Biotek Instruments, Winooski, VT, USA).

The food supplements used were Resveratrol 500 mg (Resvitále; GNC, Brookhaven, PA, USA) and Vitamin C/Ascorbic Acid tablets (C-500; GNC, Brookhaven, PA, USA). The placebos used were fabricated according to good practices for manufacturing by Carnot laboratories^®^, Mexico City, Mexico.

### 2.7. Sample Size

The sample size was determined to detect a mean difference in antioxidant markers at an alpha level of 0.05 and 80% power. The minimum required number of participants was 12 per group.

### 2.8. Statistical Analysis

Data were analyzed using GraphPad Prism version 8.0 (GraphPad Software, San Diego, CA, USA). Data are presented as mean ± standard deviation (SD). The distribution of continuous variables was assessed with the Kolmogorov–Smirnov test, and all variables were normally distributed. Within-group comparisons between baseline and post-intervention measurements were performed using a paired Student’s *t*-test. To evaluate treatment effects over time, a two-way repeated-measures analysis of variance (ANOVA) was performed, with treatment (resveratrol, resveratrol plus vitamin C, and vitamin C) as the between-subject factor, and time (baseline and 3 months) as the within-subject factor, and the treatment × time interaction was included in the model. Statistical significance was assessed for the main effects of treatment and time, as well as for their interaction. Because only two repeated measurements were obtained, the assumption of sphericity was inherently satisfied. All reported *p*-values are two-sided, and a *p*-value < 0.05 was considered statistically significant.

## 3. Results

### 3.1. Participants

**Figure 1:**
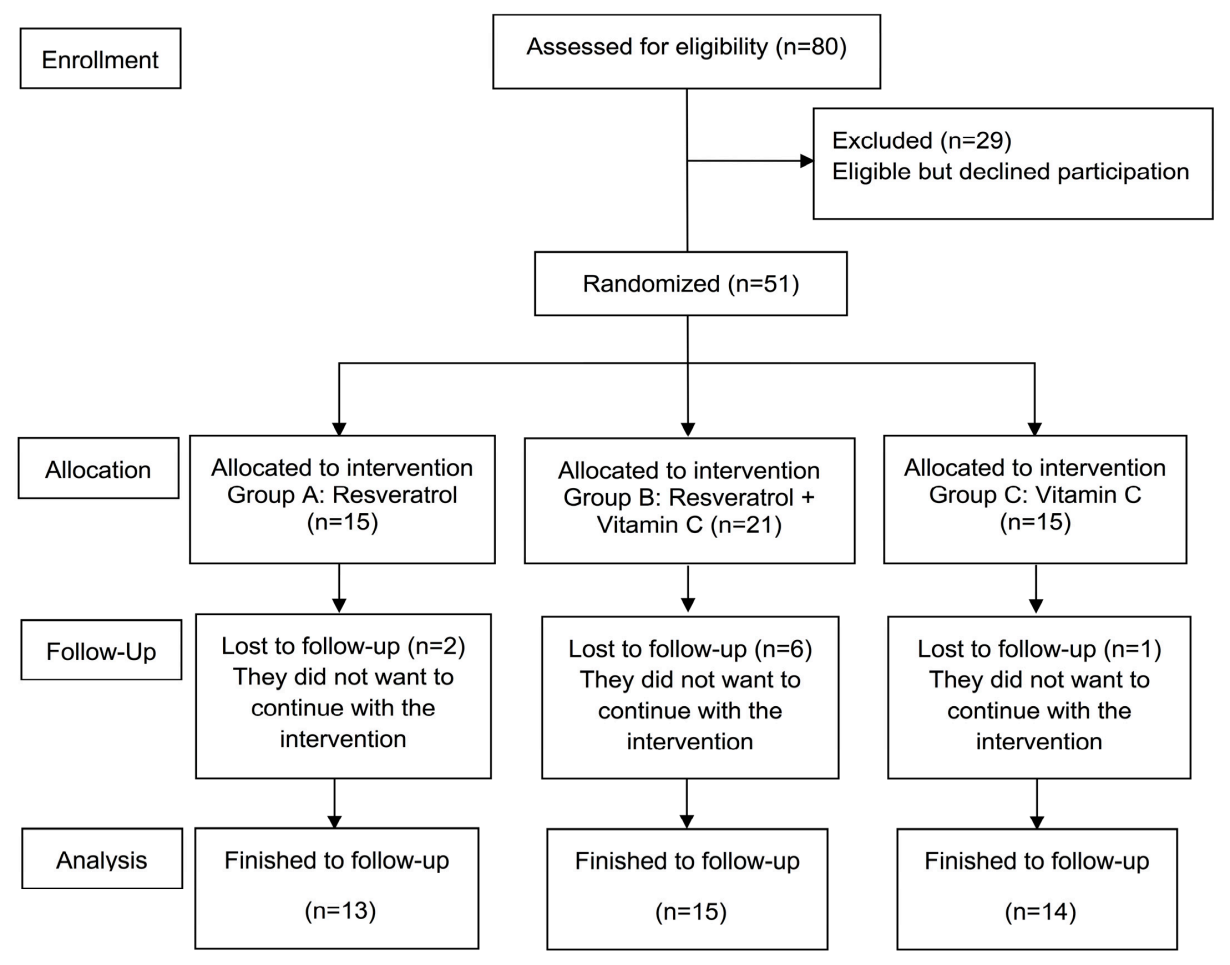
Flowchart of the participants in the study.

Participant flow and group allocation are shown in the participant flowchart, previously published as Figure 1 [19]. In brief, of the women initially assessed, 42 met eligibility criteria and were randomized to group A) Resveratrol and placebo (n = 13), group B) Resveratrol and vitamin C (n = 15), or group C) Vitamin C and Placebo (n = 14). Both patients and healthcare providers remained blinded to treatment assignment. Supplementation was taken once daily before breakfast and continued for three months.

No significant differences were observed between the basal values and the values three months after supplementation for weight, BMI, glucose, insulin, lipid profile, and uric acid within each group of the study, as shown in the previously published Table 2 [19].

### 3.2. Quantification of Oxidative Damage

**Figure 2:**
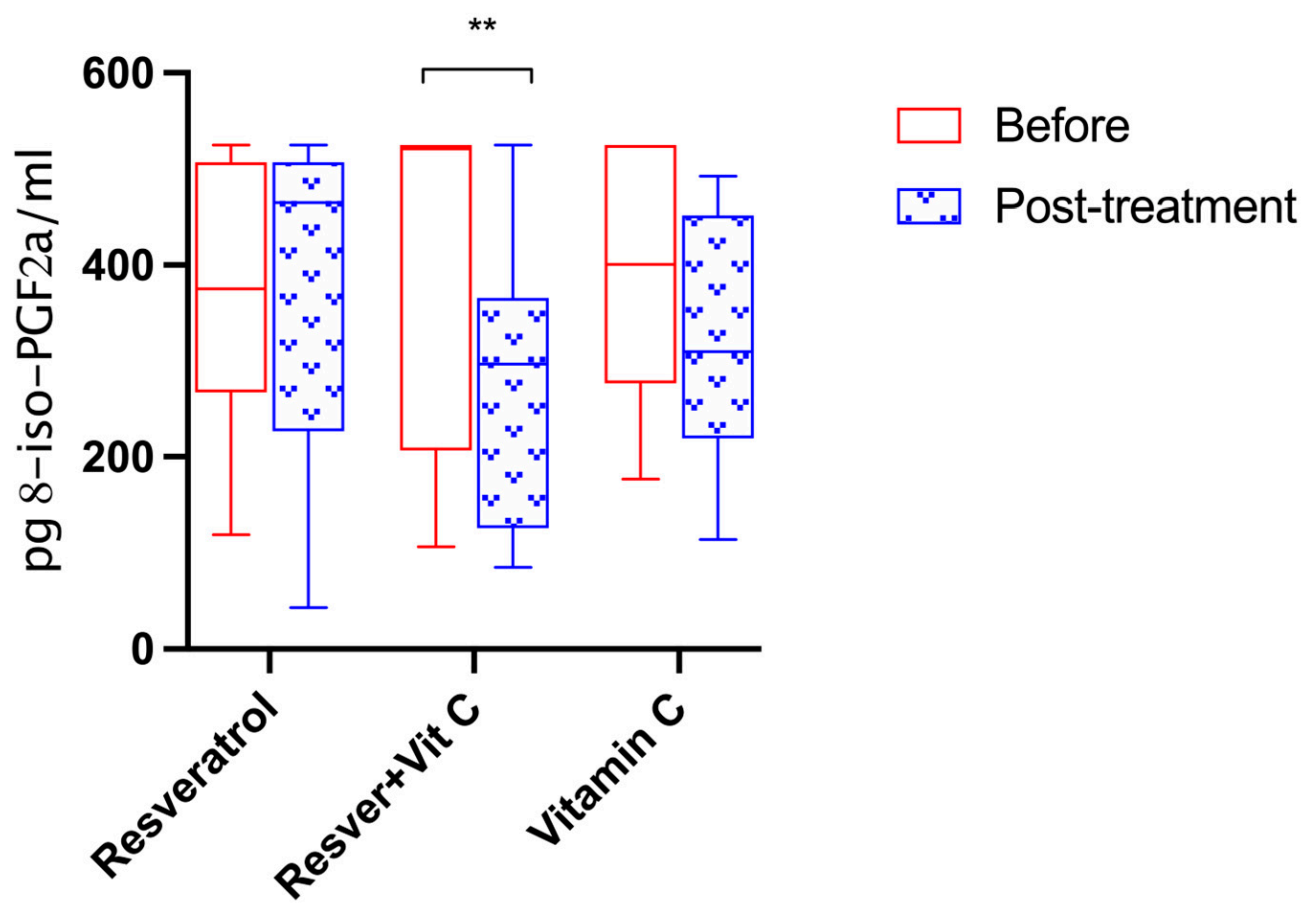
8-isoprostane levels before and after treatment (resveratrol, resveratrol + vitamin C, and vitamin C) in postmenopausal women with insulin resistance. ** *p* < 0.01.

Figure 2 shows pre- and post-treatment plasma 8-isoprostane concentrations across the three intervention groups. Two-way repeated-measures ANOVA showed no significant differences among treatment groups at the post-intervention assessment and no significant treatment × time interaction, indicating that overall changes in plasma 8-isoprostane concentrations were comparable across the three interventions. Within-group analyses showed reductions in plasma 8-isoprostane concentrations of 2%, 34%, and 19% in Groups A, B, and C, respectively. A statistically significant reduction from baseline was observed only in Group B (*p* = 0.01), whereas reductions in Group A (*p* = 0.96) and Group C (*p* = 0.12) were not statistically significant.

**Figure 3:**
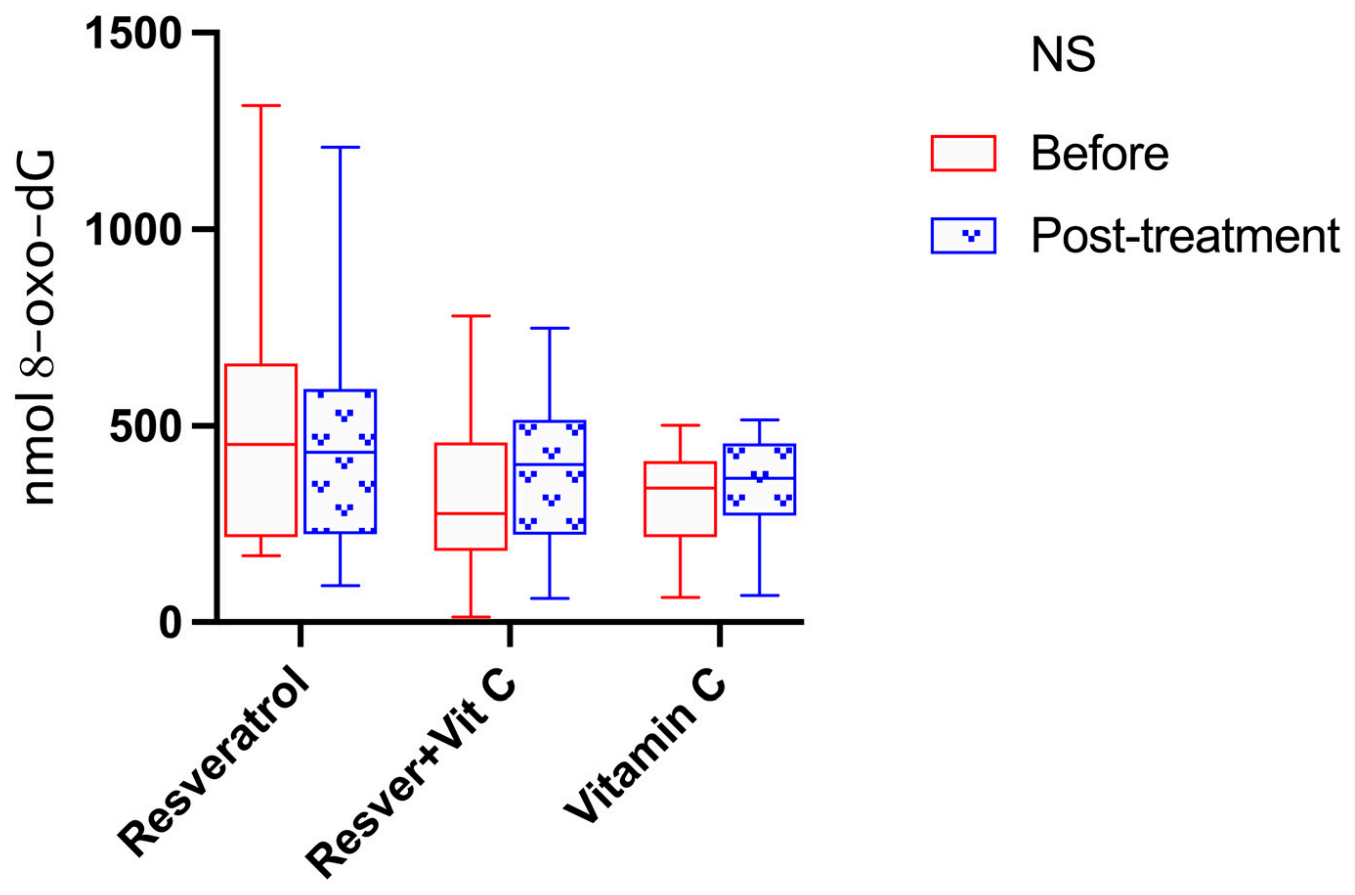
8-Oxo-dG levels before and after treatment (resveratrol, resveratrol + vitamin C, and vitamin C) in postmenopausal women with insulin resistance.

Figure 3 shows pre- and post-treatment plasma 8-oxo-dG concentrations across the three intervention groups. Two-way repeated-measures ANOVA revealed no significant differences among treatment groups and no significant treatment × time interaction, indicating that oxidative DNA damage remained unchanged throughout the study. Within-group analyses showed an 8% decrease in Group A and increases of 17% and 11% in Groups B and C, respectively. However, none of these within-group changes reached statistical significance (Group A, *p* = 0.86; Group B, *p* = 0.36; Group C, *p* = 0.46).

### 3.3. Quantification of Antioxidant Defense

**Figure 4:**
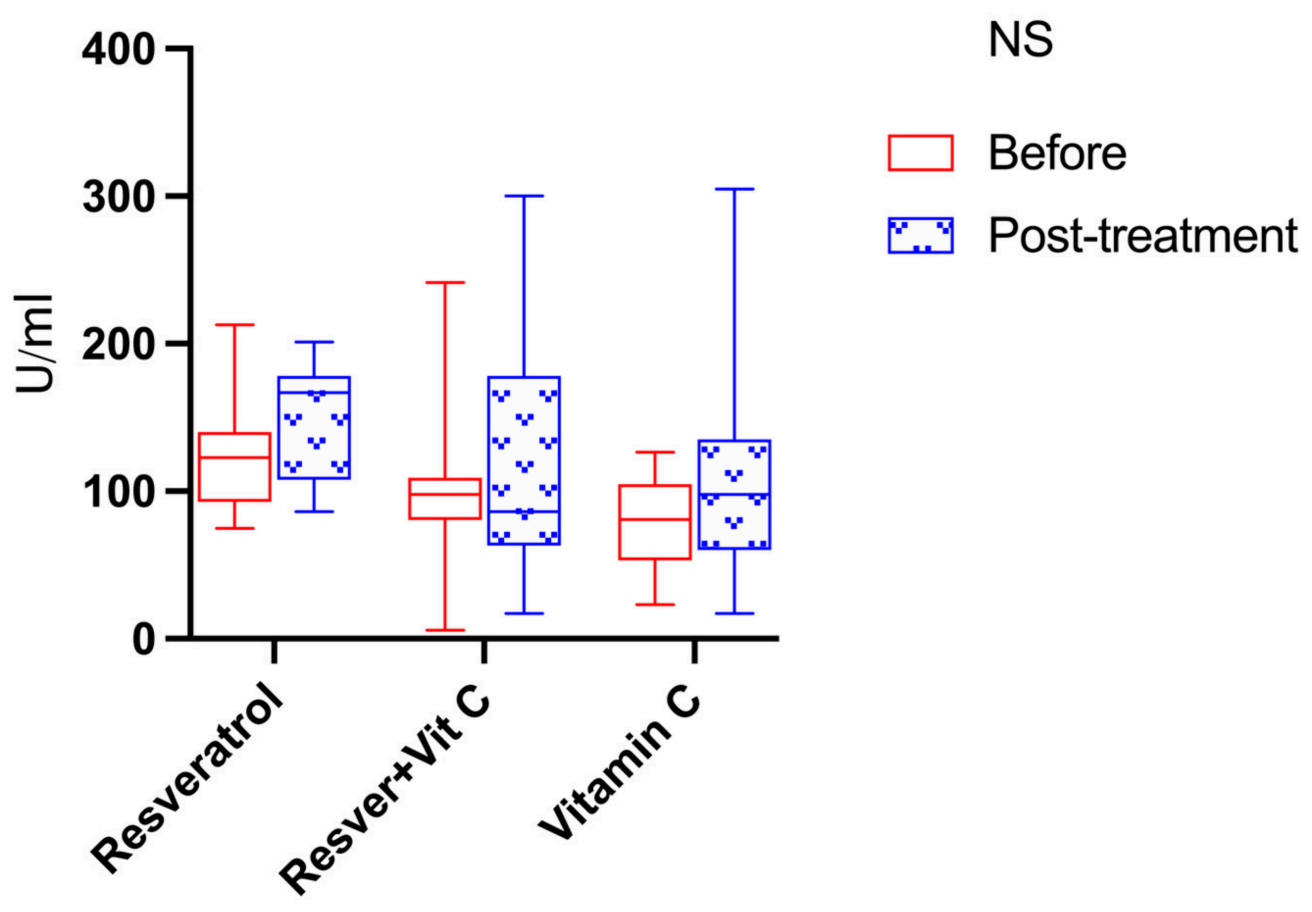
SOD enzyme activity before and after treatment (resveratrol, resveratrol + vitamin C, and vitamin C) in postmenopausal women with insulin resistance.

Figure 4 shows SOD activity before and after the intervention in the three treatment groups. Two-way repeated-measures ANOVA revealed no significant differences among treatment groups and no significant treatment × time interaction, indicating that SOD activity remained comparable across the three interventions throughout the study period.

Within-group analyses showed increases in SOD activity of 21%, 30%, and 35% in Groups A, B, and C, respectively, after the 3-month intervention. However, none of these changes reached statistical significance (Group A, *p* = 0.10; Group B, *p* = 0.63; Group C, *p* = 0.42).

**Figure 5:**
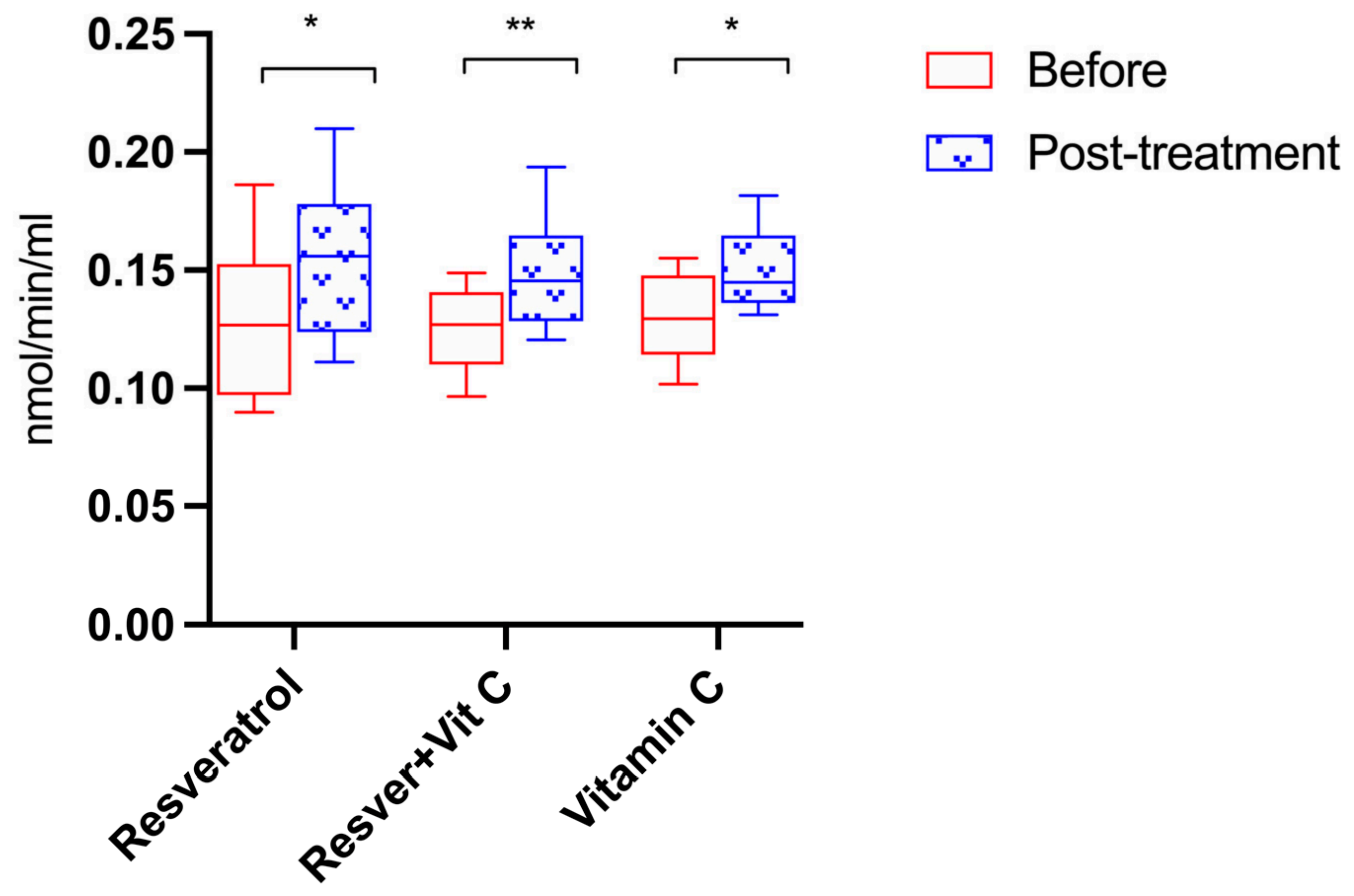
CAT enzyme activity before and after treatment (resveratrol, resveratrol + vitamin C, and vitamin C) in postmenopausal women with insulin resistance. * *p* < 0.05, ** *p* < 0.01.

Figure 5 presents CAT activity before and after the 3-month intervention in the three treatment groups. Two-way repeated-measures ANOVA detected no significant between-group differences and no significant treatment × time interaction. Within-group analyses demonstrated significant increases in CAT activity relative to baseline in all three groups, corresponding to increases of 21%, 18%, and 12% in Groups A, B, and C, respectively (Group A, *p* = 0.03; Group B, *p* = 0.0086; and Group C, *p* = 0.0241).

**Figure 6:**
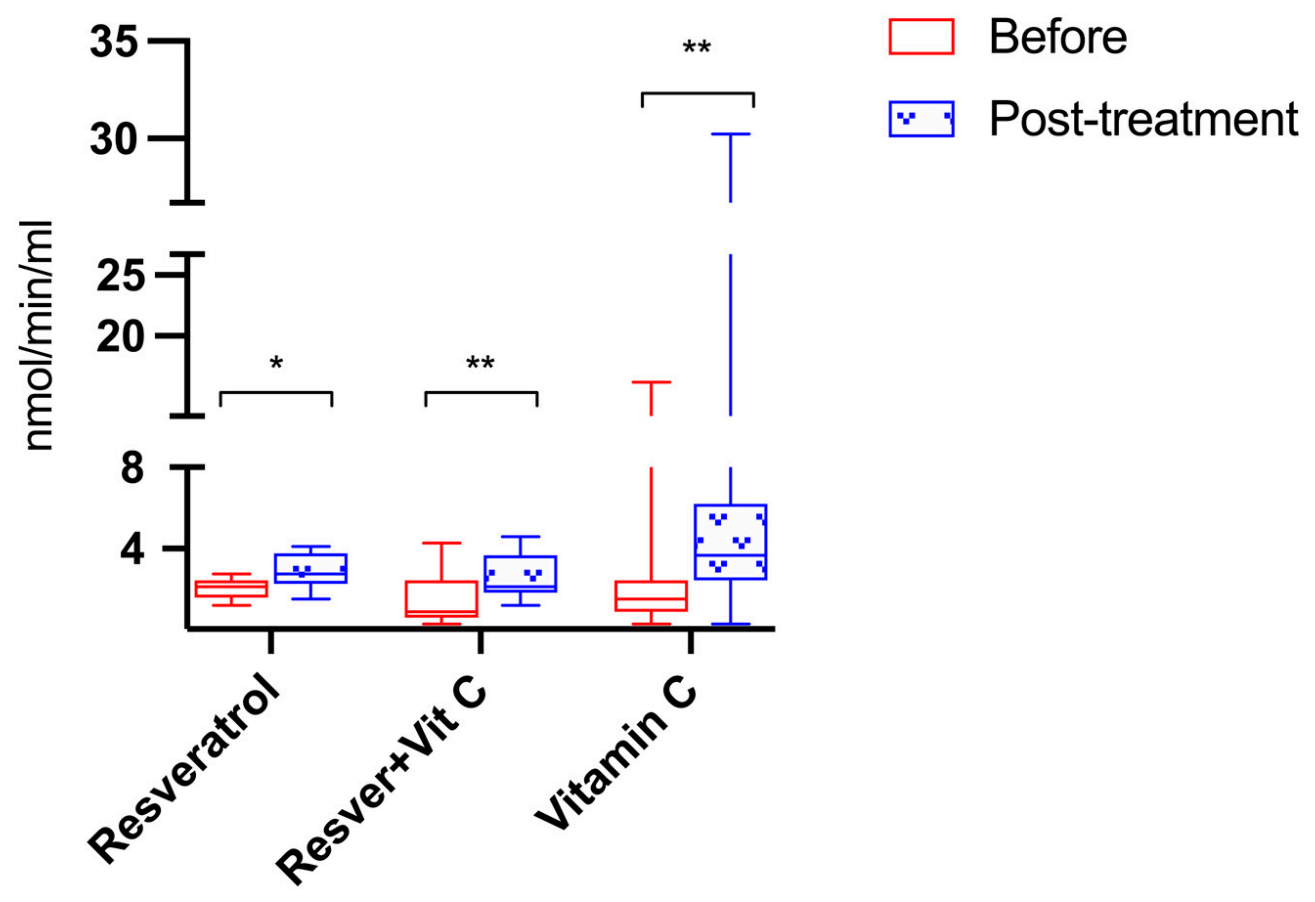
GR enzyme activity before and after treatment (resveratrol, resveratrol + vitamin C, and vitamin C) in postmenopausal women with insulin resistance. * *p* < 0.05, ** *p* < 0.01.

Figure 6 presents GR activity before and after the 3-month intervention in the three treatment groups. Two-way repeated-measures ANOVA revealed no significant between-group differences and no significant treatment × time interaction. Within-group analyses showed significant increases in GR activity relative to baseline in all three groups. GR activity increased by 37%, 84%, and 150% in Groups A, B, and C, respectively (Group A, *p* = 0.0176; Group B, *p* = 0.0071; and Group C, *p* = 0.0028).

**Figure 7:**
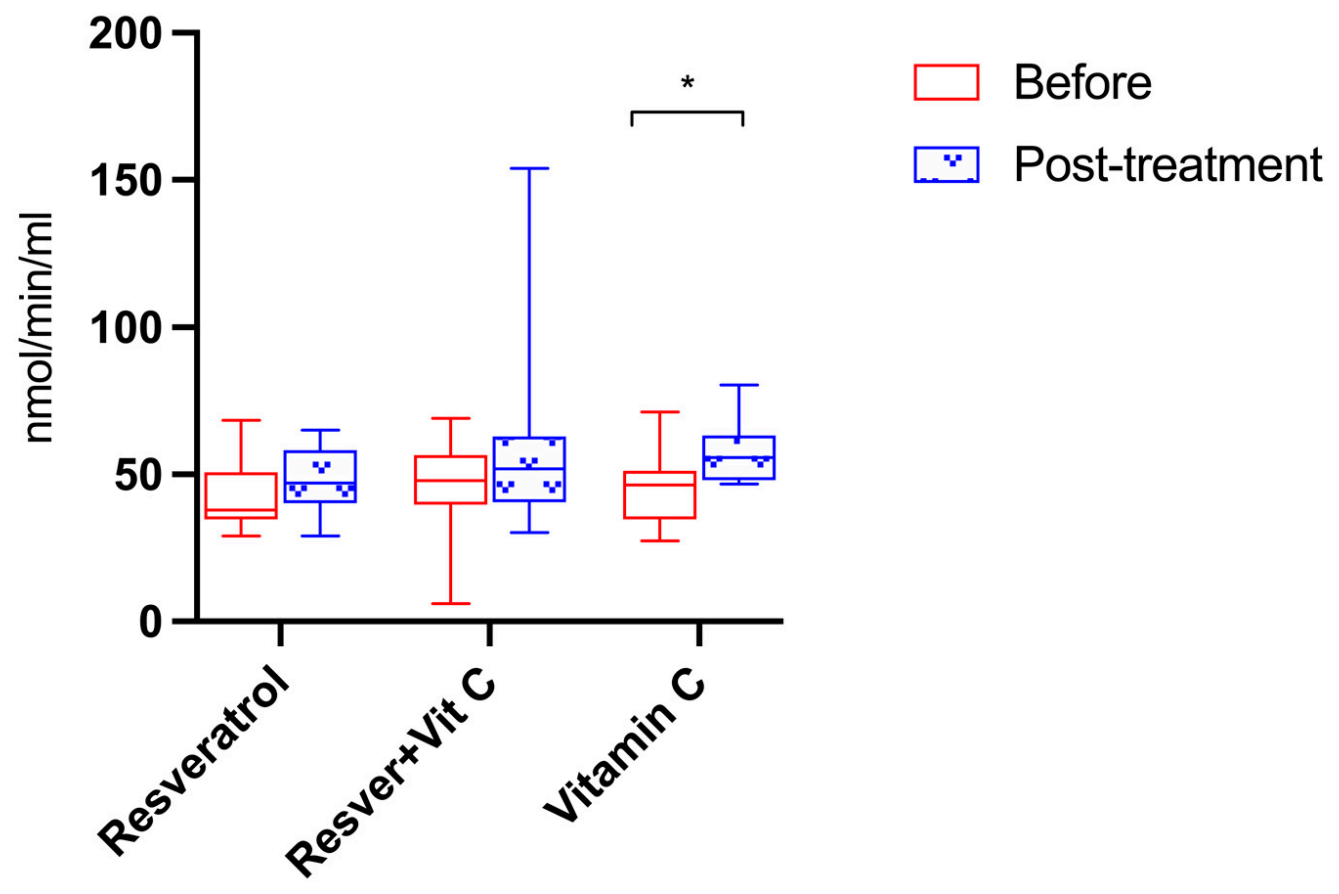
GPx enzyme activity before and after treatment (resveratrol, resveratrol + vitamin C, and vitamin C) in postmenopausal women with insulin resistance. * *p* < 0.05.

Figure 7 presents GPx activity before and after the 3-month intervention in the three treatment groups. Two-way repeated-measures ANOVA detected no significant between-group differences and no significant treatment × time interaction. Within-group analyses demonstrated increases in GPx activity relative to baseline of 13%, 22%, and 25% in Groups A, B, and C, respectively. These changes reached statistical significance only in Group C (*p* = 0.019), whereas the increases observed in Group A (*p* = 0.09) and Group B (*p* = 0.64) were not statistically significant.

## 4. Discussion

The present pilot randomized, double-blind clinical trial evaluated the effects of antioxidant supplementation on oxidative stress biomarkers in insulin-resistant postmenopausal women. Significant within-group increases in CAT, GR, and GPx activities were observed, along with a reduction in plasma 8-isoprostane concentrations in one intervention group. In contrast, oxidative DNA damage assessed by 8-Oxo-dG remained unchanged. However, no significant between-group differences or treatment × time interactions were detected, indicating that the present study does not demonstrate the superiority of one antioxidant regimen over another. These findings should therefore be considered preliminary and interpreted within the context of a pilot clinical trial with a relatively small sample size.

The observed changes are consistent with the current understanding of the metabolic alterations associated with menopause and insulin resistance. Estrogen deficiency promotes mitochondrial dysfunction, endothelial impairment, chronic low-grade inflammation, and excessive ROS [21]. Simultaneously, insulin resistance amplifies oxidative stress through increased mitochondrial electron leakage, activation of NADPH oxidase, accumulation of advanced glycation end products, and persistent activation of inflammatory pathways such as nuclear factor-κB (NF-κB) [21]. Together, these mechanisms contribute to redox imbalance and increase the risk of cardiovascular disease, metabolic syndrome, type 2 diabetes, and other age-related disorders. Although the present study did not evaluate the molecular pathways underlying the observed biochemical changes, our findings support the rationale for further investigating antioxidant interventions as a strategy to improve redox homeostasis in this population [21,22].

Among the antioxidant biomarkers evaluated, the most consistent biochemical response was the increase in CAT and GR activities following antioxidant supplementation. CAT is a key enzymatic defense against hydrogen peroxide accumulation. In contrast, GR regenerates reduced glutathione (GSH), thereby preserving the intracellular GSH/GSSG balance required for GPx activity and protection against oxidative damage [1,23]. The observed increases in these enzymes suggest an enhancement in endogenous antioxidant defenses, rather than a simple free radical scavenging effect.

Although the present study was not designed to investigate intracellular signaling pathways, the observed enzymatic changes are consistent with mechanisms previously described for both resveratrol and vitamin C. Experimental evidence indicates that resveratrol activates SIRT1/AMPK signaling, enhances mitochondrial biogenesis, and promotes Nrf2-mediated transcription of antioxidant genes, including CAT, GPX1, GSR, HO-1, and NQO1 [22,24,25]. Vitamin C may further contribute by directly scavenging reactive oxygen species, regenerating oxidized vitamin E, preserving intracellular glutathione, and maintaining the cellular redox environment required for optimal antioxidant enzyme activity [26,27,28]. Because these pathways were not directly evaluated, they should be regarded as biologically plausible mechanisms supported by previous experimental studies, rather than direct evidence generated by the present trial.

One of the principal biochemical findings of this study was the reduction in plasma 8-isoprostane concentrations. F2-isoprostanes are among the most reliable biomarkers of lipid peroxidation because they are generated through free radical-mediated oxidation of arachidonic acid independently of cyclooxygenase activity [1]. Elevated circulating 8-isoprostane concentrations have been consistently associated with obesity, insulin resistance, metabolic syndrome, endothelial dysfunction, and increased cardiovascular risk [27,29]. Although no significant between-group differences were observed, the reduction detected in one intervention group is consistent with previous reports showing that antioxidant interventions can attenuate lipid peroxidation and improve redox homeostasis under conditions of increased oxidative stress [24,25,26,27,28,29,30].

In contrast to the changes observed in lipid peroxidation, 8-Oxo-dG concentrations remained unchanged following antioxidant supplementation. Several factors may explain this finding. Oxidative DNA lesions accumulate over prolonged periods and may require longer interventions before measurable reductions become evident. Moreover, circulating 8-Oxo-dG concentrations are influenced not only by ROS generation, but also by the efficiency of DNA repair mechanisms, particularly the base excision repair pathway [8]. In addition, the relatively small sample size of this pilot trial may have limited the statistical power to detect modest changes. Therefore, the absence of significant changes in 8-Oxo-dG should not be interpreted as evidence of a lack of biological activity, but rather as an indication that different oxidative compartments may respond differently to antioxidant interventions. This interpretation is consistent with the observed improvement in antioxidant enzyme activities and the reduction in lipid peroxidation, which may precede measurable changes in oxidative DNA damage.

The enzymatic responses observed in the present study are broadly consistent with previous experimental and clinical evidence, although the magnitude of the response varied among antioxidant enzymes. CAT, GR, and GPx activities increased significantly within one or more intervention groups, whereas SOD showed only a modest, non-significant increase. Similar variability has been reported in previous studies evaluating resveratrol or vitamin C supplementation, likely reflecting differences in dosage, intervention duration, baseline oxidative status, and study populations [19,22,23,24,25,26,27,28,29,30,31]. Experimental studies have also suggested that SOD activity may be more tightly regulated than downstream antioxidant enzymes and may require longer exposure to antioxidant compounds before measurable changes become apparent. Furthermore, SOD activity depends on the availability of trace elements such as copper, zinc, and manganese, factors that were not evaluated in the present study [1,31,32].

From a clinical perspective, these findings support the concept that oxidative stress is a central mechanism linking menopause, insulin resistance, endothelial dysfunction, and cardiovascular disease. Although antioxidant supplementation should not replace established lifestyle interventions such as regular physical activity, healthy dietary patterns, and weight management, the present findings suggest that antioxidant supplementation may help improved selected biomarkers of redox homeostasis in metabolically unhealthy postmenopausal women. However, because no significant between-group differences were observed and a placebo-only control group was not included, these biochemical improvements should be interpreted as preliminary. Larger, adequately powered placebo-controlled trials are needed to determine whether these changes translate into meaningful reductions in cardiometabolic risk, diabetes progression, or healthy aging.

Several limitations should be considered when interpreting the present findings. First, this was a pilot randomized clinical trial with a relatively small sample size, which may have limited the statistical power to detect modest differences among intervention groups, particularly for biomarkers such as 8-Oxo-dG and SOD. Consistent with this, although significant within-group changes were observed for several antioxidant enzymes, the two-way repeated measures ANOVA did not identify significant between-group differences or treatment × time interactions, precluding conclusions regarding the superiority of any antioxidant regimen. Second, the intervention period was limited to three months and therefore may not have been sufficient to detect longer-term changes in oxidative DNA damage or sustained adaptations in redox homeostasis. Third, a placebo-only control group was not included. Following recommendations from the Institutional Research and Ethics Committee, all participants received at least one antioxidant intervention because prolonged placebo administration in women with documented insulin resistance and increased oxidative stress was considered ethically inappropriate. Although this design prevented comparison with an untreated control group, it allowed evaluation of different antioxidant strategies under ethically acceptable conditions. Fourth, the study included only early postmenopausal Mexican women with insulin resistance, limiting the generalizability of the findings to other ethnic populations, healthy postmenopausal women, or women with established diabetes mellitus. Finally, dietary antioxidant intake, physical activity, inflammatory cytokines, mitochondrial function, intracellular glutathione homeostasis (GSH/GSSG ratio), and components of the SIRT1/AMPK/Nrf2 signaling pathway were not assessed. Consequently, the molecular mechanisms discussed should be interpreted as biologically plausible hypotheses supported by previous evidence, rather than mechanisms directly demonstrated in this clinical trial.

Despite these limitations, the present study has several important strengths. To our knowledge, this is one of the first randomized, double-blind clinical trials to simultaneously evaluate a comprehensive panel of enzymatic antioxidant defenses (SOD, CAT, GR, and GPx) together with biomarkers of lipid peroxidation (8-isoprostane) and oxidative DNA damage (8-Oxo-dG) in insulin-resistant postmenopausal women receiving antioxidant supplementation. The randomized design, high treatment adherence, rigorous biochemical assessment, and simultaneous evaluation of multiple components of redox homeostasis provide a comprehensive characterization of the antioxidant response in this clinically relevant population. These findings provide valuable evidence on the biochemical effects of antioxidant supplementation in early postmenopausal women and lay the foundation for larger, adequately powered randomized clinical trials designed to determine the comparative efficacy of different antioxidant strategies.

Future multicenter trials with larger sample sizes, longer follow-up, and the integration of transcriptomic, metabolomic, and mitochondrial biomarkers will be essential to determine whether activation of the SIRT1–AMPK–Nrf2 signaling network translates into sustained improvements in metabolic health and healthy aging among postmenopausal women.

## 5. Conclusions

This pilot randomized, double-blind clinical trial provides preliminary evidence that antioxidant supplementation may enhance selected components of the endogenous antioxidant defense system in insulin-resistant postmenopausal women. Significant within-group improvements were observed in CAT, GR, and GPx activities, along with reduced plasma 8-isoprostane concentrations in one intervention group. Oxidative DNA damage, assessed by 8-Oxo-dG, remained unchanged.

Importantly, no significant between-group differences or treatment × time interactions were observed, so the present findings do not support the superiority of one antioxidant regimen over another. In addition, the absence of a placebo-only control group limits the ability to attribute the observed biochemical changes exclusively to the interventions. These findings should be considered hypothesis-generating rather than definitive. Larger, adequately powered, placebo-controlled randomized clinical trials with longer follow-up and mechanistic biomarkers are warranted to confirm these preliminary observations and clarify the role of antioxidant supplementation in improving redox homeostasis during postmenopause.

## Data Availability

The datasets generated during and/or analyzed during the present study are not publicly available, but are available from the corresponding author on reasonable request.
